# Orphan Nuclear Receptor NR4A1 Promotes Proliferation and Osteogenic Differentiation of Valvular Interstitial Cells through Activation of CCND2

**DOI:** 10.7150/ijbs.122863

**Published:** 2026-02-26

**Authors:** Qiang Shen, Chao Zhang, Chen Jiang, Zongtao Liu, Lin Fan, Hanshen Luo, Xingyu Qian, Zhengfeng Fan, Dingyi Yao, Nianguo Dong, Jiawei Shi

**Affiliations:** 1 Department of Cardiovascular Surgery, Union Hospital, Tongji Medical College, Huazhong University of Science and Technology, Wuhan, China.; 2 Fuwai Hospital, Chinese Academy of Medical Sciences, China.

**Keywords:** Calcific aortic valve disease, nuclear receptor, lysine demethylase 6B, cyclin D2, cellular proliferation, osteogenic differentiation.

## Abstract

Calcific aortic valve disease (CAVD), the most common human valve disease on a global scale, ranks and persists as an unaddressed clinical challenge. This is primarily attributed to the absence of efficacious pharmacological approaches. The Nuclear Receptor Subfamily 4 Group A Member 1 (NR4A1), intricately associated with the pathogenesis of multiple cardiovascular diseases, has emerged as a pivotal target for the diagnosis and treatment of numerous ailments. However, the specific molecular mechanisms and the functional significance of NR4A1 in the pathogenesis of CAVD are yet to be comprehensively elucidated. By performing in-depth analyses on human aortic valve tissues and carrying out functional investigations using primary valvular interstitial cells (VICs), we were able to demonstrate that NR4A1 significantly facilitated cellular proliferation and intensifies the osteogenic differentiation process of VICs. Evidently, this is reflected in the elevated expression of key osteogenic markers, namely runt-related transcription factor 2 (RUNX2) and alkaline phosphatase (ALP). Mechanistically, the pro-calcific effects were achieved via NR4A1-dependent modulation of the cell cycle regulatory protein Cyclin D2 (CCND2). Significantly, *in vivo* investigations employing ApoE^-/-^ mice maintained on a high-fat Western diet demonstrated that pharmacological suppression of NR4A1 efficiently mitigated the advancement of aortic valve calcification. These discoveries not merely determine NR4A1 to be a crucial modulator in cellular proliferation, thereby accelerating valvular calcification, but also present compelling evidence advocating for targeting NR4A1 may represent a potential therapeutic strategy for CAVD.

## Introduction

Calcific aortic valve disease (CAVD), a degenerative disorder, is typically characterized by aortic valve stenosis and sclerosis and is associated with a high morbidity and mortality rate 1. A significant rise in the incidence of CAVD is observed as age progresses. To date, the only therapeutic option for patients with severe symptomatic CAVD is aortic valve replacement 2. Owing to the escalating demand for surgical treatments in the aging population, a full - fledged understanding of the molecular and cellular mechanisms fueling CAVD pathogenesis is indispensable. This is necessary to drive the development of targeted pharmacological therapies capable of halting or reversing the advancement of the disease.

The NR4A family, an ensemble of orphan nuclear receptors encompassing NR4A1 (NUR77), NR4A2 (NURR1), and NR4A3 (NOR1), represents a distinct subgroup of transcription factors (TFs) 3. These TFs are crucial for modulating tissue responses 3. Studies have reported that the NR4A family of orphan nuclear receptors is associated with atherosclerosis and vascular calcification, whose etiologies share similarities with CAVD 4. Among them, NR4A1 has been a subject of controversy regarding its role in atherosclerosis 5-7. Notably, Zhu et al. demonstrated that NR4A1 promotes vascular calcification by inhibiting mitophagy 8. Additionally, Ma et al. reported that NR4A1 knockout ameliorates disorders of glucose and lipid metabolism, mitochondrial dysfunction, and pathological cardiac remodeling 9. Yet, our understanding of the particular function of NR4A1 in CAVD is scant, and this area is in need of further in-depth study.

The process of cellular proliferation holds a central position in the development and the advancement course of cardiovascular diseases 10-12. The proliferation of valvular interstitial cells (VICs) and the over-generation of extracellular matrix (ECM) proteins jointly drive the development of valvular fibrosis. This pathological process, in turn, induces alterations in the mechanical traits manifested by aortic valve leaflets 13-15. A number of studies have recognized the effect that proliferative mediators, among which are BMP-2, IL-1, and TGF-β1, exert on the proliferation of valvular cells 16-18. Cyclin D2 (CCND2), a cell cycle-related protein, has been shown to promote cellular proliferation 19. Hence, a more profound comprehension of the function of CCND2 in the osteogenic differentiation of VICs might offer valuable perspectives for formulating efficacious therapeutic approaches against CAVD.

The present study reveals that NR4A1 expression is markedly increased in aortic valves that have undergone calcification. *In vitro*, NR4A1 promotes the proliferation of VICs by upregulating the cell cycle-related protein CCND2, thereby facilitating the osteogenic differentiation process of VICs. Moreover, we determined that the increase in NR4A1 levels is brought about by the demethylation function of Jumonji Domain Containing 3 (JMJD3). These results imply that the targeting of NR4A1 or CCND2 could potentially serve as an innovative medical approach for the management of CAVD.

## Methods

### Human samples and ethical principles

Normal aortic valves utilized in the control group were procured from patients who underwent heart transplantation for heart failure and exhibited no signs of calcification. In contrast, at Wuhan Union Hospital's Department of Cardiovascular Surgery, calcified aortic valves were obtained from patients who had undergone surgical aortic valve replacement. All patients furnished written informed consent. Moreover, the study obtained ethical clearance (No: UHCT-IEC-SOP-016-03-01) from the Ethics Committee of Union Hospital and Tongji Medical College, Huazhong University of Science and Technology in Wuhan, China.

### Isolation of RNA and quantitative PCR analysis

Total RNA was extracted using the RNA Isolation Kit (Cat. No. RC112 - 01, Vazyme). Subsequently, for the quantitative real-time polymerase chain reaction (qPCR), the reaction mixture was prepared with the 2x SYBR Green qPCR Master Mix (Cat. No. Q711 - 02, Vazyme) following the provided protocols. The polymerase chain reaction was then carried out on a StepOne Plus thermal cycler (Applied Biosystems, USA). The primer sequences were presented in **Supplementary Table S1**.

### Western blot analysis

Protein extracts derived from either aortic valve tissues or VICs were subjected to immunoblot analysis. Specifically, the tissues or cells were lysed on ice for 10 minutes. After lysis, the lysate was centrifuged at high speed. Subsequently, the supernatant was carefully aspirated, added to the loading buffer, and then subjected to boiling. Then, the expression of the target proteins was evaluated via Western blotting (WB).

### RNA isolation and RNA-sequencing analysis

Total RNA was extracted from VICs by means of the RNAsimple Total RNA Kit from TIANGEN Biotech (China). Subsequently, the RNA samples were dispatched to Ouyi Biotechnology (Shanghai, China) for RNA sequencing analysis.

### Immunohistochemistry staining

The aortic valve tissues were fixed in paraformaldehyde and then embedded in paraffin. After the paraffin sections were dewaxed, rehydrated and subjected to other treatments, they were incubated with the primary antibody overnight at 4 degrees Celsius. Subsequently, they were cultivated with the secondary antibody conjugated with horseradish peroxidase (HRP).

### Immunofluorescence staining

After dewaxing, the paraffin sections were fixed with 4% paraformaldehyde for 20 minutes. Subsequently, they were permeabilized with PBS containing 0.1% Triton X-100 for 15 minutes. After being washed three times with PBS, the sections were blocked for 1 hour. After washing following overnight incubation with the primary antibody, samples were incubated with the secondary antibody at room temperature. Finally, 4',6-diamidino-2-phenylindole (DAPI) was added for nuclear staining. Throughout the entire process of incubation with the secondary antibody and during the 10 - minute DAPI staining procedure, stringent light-avoidance measures were strictly implemented.

### Isolation and culture of VICs

VICs were isolated by collecting aortic valves from patients who underwent heart transplantation. In the initial phase, the tissue specimens underwent three consecutive irrigations with PBS to prepare them for subsequent procedures. In the subsequent step, the tissues were carefully minced into minute fragments. Thereafter, digestion was initiated by exposing the minced tissues to a 1 mg/mL Collagenase type I, which was maintained at 37°C for a period spanning 12 h. Subsequently, to eliminate any undigested substances, the digested tissue was carefully filtered through a nylon mesh. After isolation, the isolated VICs were propagated in high-glucose Dulbecco's Modified Eagle Medium (DMEM) to which a blend consisting of 10% fetal bovine serum (FBS) had been added. These cells were cultured in a CO₂ incubator under standard conditions. For all subsequent experiments, VICs at passage 3 were selected and employed.

### Adenovirus-mediated overexpression

Recombinant adenoviral vectors engineered for the purpose of encoding the designated genes were rigorously developed and procured from WZ Biosciences Inc., a reputable biotech firm located in Shandong, China. To initiate the adenoviral infection process, the cells seeded in 6- well plates were exposed to adenovirus at a multiplicity of infection (MOI) of 70. After a 48-hour adenoviral transduction period, subsequent experimental manipulations were carried out. These manipulations encompassed induction with osteogenic medium (OM) and treatment under other specified experimental conditions.

### CCK-8 assay

cells were seeded in 96-well plates with 100 μL of culture medium per well. Subsequently, the culture medium was replaced with a medium containing 2% fetal bovine serum (FBS) to induce cell starvation for 12 h. Thereafter, different doses of DIM-C were added. After 3 days of inhibitor intervention, 10 μL of CCK-8 reagent (Beyotime, C0037) was added to the culture medium, and the absorbance at 450 nm was measured.

### Alkaline phosphatase staining

For alkaline phosphatase (ALP) staining, cells were fixed with 4% paraformaldehyde for 15 minutes on day 7 post-intervention, followed by chromogenic staining using a BCIP/NBT alkaline phosphatase detection kit in accordance with the manufacturer's protocols. For ALP activity measurement, VICs were first incubated with Alamar Blue for 1 h to quantify cell number. The cells were then lysed and incubated with p-nitrophenyl phosphate in an alkaline buffer (containing Na₂CO₃, NaHCO₃ and MgCl₂, pH ≈ 10) for 20 min. The absorbance at 405 nm (A405) was recorded and ALP activity was normalized to the corresponding Alamar Blue fluorescence value 20.

### Experimental animals and protocols

ApoE^-/-^ mice were procured from Wuhan Shulaibao Biotechnology Co., Ltd. A total of twenty 8-week-old male ApoE^-/-^ mice were randomly assigned into two separate groups, with each group consisting of 10 mice. Specifically, the first group was designated as the control group. Mice in this group were fed a Western-style diet and concurrently administered corn oil. In contrast, the second group, referred to as the experimental group, was also provided with the identical Western-style diet. However, the mice in the experimental group were orally gavaged with DIM-C-pPhCO₂Me (DIM-C) at a dosage of 40 mg/kg. The administration frequency was twice a week, and the treatment duration was 24 weeks. At the conclusion of the 24-week period, under 2.5% isoflurane anesthesia, cardiovascular hemodynamic parameters were evaluated via transthoracic echocardiography. After the echocardiographic assessment was completed, the mice were humanely euthanized by intraperitoneal injection of pentobarbital sodium. After thorough flushing with physiological saline, the hearts were excised and fixed with 4% paraformaldehyde. The research protocol was approved by the Institutional Animal Care and Use Committee of Tongji Medical College, Huazhong University of Science and Technology ([2023] IACUC No: 4470).

### Small interfering RNA (siRNA)-mediated silencing

For the modulation of NR4A1, CCND2, and JMJD3 expression in VICs, cells were cultured until reaching 70 80% confluence. Subsequently, After the cells were starved for 12 h, siRNAs were transfected into the cells using Lipofectamine 3000. The culture medium was replaced 8 hours post - transfection. The siRNAs designed to target NR4A1, CCND2, and JMJD3 were expertly engineered and fabricated by RiboBio. The detailed targeting sequences of these siRNAs are presented in **Supplementary Table S1**.

### Analysis of single-cell RNA sequencing datasets

We utilized the previously published single-cell dataset from the project PRJNA562645 for subsequent analyses. This dataset encompasses 6 aortic valve specimens, which are further divided into 2 healthy samples and 4 samples from patients diagnosed with CAVD. To dissect the dataset, we employed the "Seurat" R package to carry out a series of operations, including data embedding, visualization, clustering, and principal component annotation. Additionally, gene-gene correlation analysis was conducted using the Rmagic software package.

### Antibodies and reagents

The following are the sources of all antibodies and reagents used in this study: runt-related transcription factor 2 (RUNX2) (CST, 8486, dilution ratio of 1:1000 for WB), RUNX2 (Santa Cruz, sc-390715, dilution ratio of 1:100 for Immunofluorescence staining), alkaline phosphatase (ALP) (MAB29092, R&D, dilution ratio of 1:1000 for WB), GAPDH (Proteintech, 60004-1-Ig, dilution ratio of 1:10000 for WB), NR4A1(CST, 3960, dilution ratio of 1:1000 for WB, dilution ratio of 1:100 for Immunofluorescence staining and immunohistochemistry staining), NR4A1 (Proteintech, 12235-1-AP, ChIP), CCND2 (abclonal, A13284, dilution ratio of 1:1000 for WB), JMJD3 (Proteintech, 55354-1-AP, dilution ratio of 1:1000 for WB, dilution ratio of 1:100 for Immunofluorescence staining), H3K27me3 (Proteintech, 91403, dilution ratio of 1:1000 for WB), CD31 (Proteintech, 66065-2-Ig, dilution ratio of 1:100 for Immunofluorescence staining), Cy3-labeled anti-mouse IgG (GB21401, Servicebio, dilution ratio of 1:200 for Immunofluorescence staining and Immunohistochemistry staining), Cy3-labeled anti-rabbit IgG (GB21403, Servicebio, dilution ratio of 1:200 for Immunofluorescence staining), 488-labeled anti-mouse IgG (GB25301, Servicebio, dilution ratio of 1:200 for Immunofluorescence staining), 488-labeled anti-rabbit IgG (GB25303, Servicebio, dilution ratio of 1:200 for Immunofluorescence staining). Chemicals used in the study were procured from different sources: Csn-B (MCE, HY-N2148), DIM-C-pPhCO2Me (MCE, HY-112056), GSK-J1 (MCE, HY-15648), GSK2879552 (MCE, HY-18632), Daminozide (MCE, HY-13643), CPI-455 (MCE, HY-100421).

### Chromatin immunoprecipitation (ChIP) assay

Chromatin immunoprecipitation (ChIP) assays were performed using the ChIP Assay Kit (Beyotime, Cat: P2078) following the manufacturer's instructions. Briefly, first, for sample sonication: formaldehyde cross-linking was conducted initially, followed by adding Glycine Solution. Then, cells were washed with PBS containing PMSF, scraped, centrifuged, lysed, incubated, and sonicated. Next, for chromatin immunoprecipitation: the product from the sonication step was centrifuged, 20 μL was taken as input, and the remainder was mixed with Protein A+G Agarose/Salmon Sperm DNA. Primary antibody was added and incubated overnight, after which Protein A+G Agarose/Salmon Sperm DNA was added again and incubated for 1 h before centrifugation. Finally, for PCR amplification of target gene sequences: the product from the immunoprecipitation step was mixed with 250 μL Elution buffer by vortexing, centrifuged, NaCl was added to reverse cross-linking, and then real-time PCR was performed. The primer sequences were presented in **Supplementary Table S1**.

### Statistics analysis

Data were analyzed using GraphPad Prism 9 (GraphPad Software, Inc., La Jolla, CA, USA) and presented as means ± SD. For statistical comparisons: the unpaired t-test was used for two-group comparisons; one-way analysis of variance (ANOVA) was employed for multiple-group comparisons, followed by Tukey's post hoc test to determine pairwise significance. Statistical significance was defined as P < 0.05.

## Results

### Marked upregulation of NR4A1 in human calcified aortic valves (CAVs)

**Figure 1:**
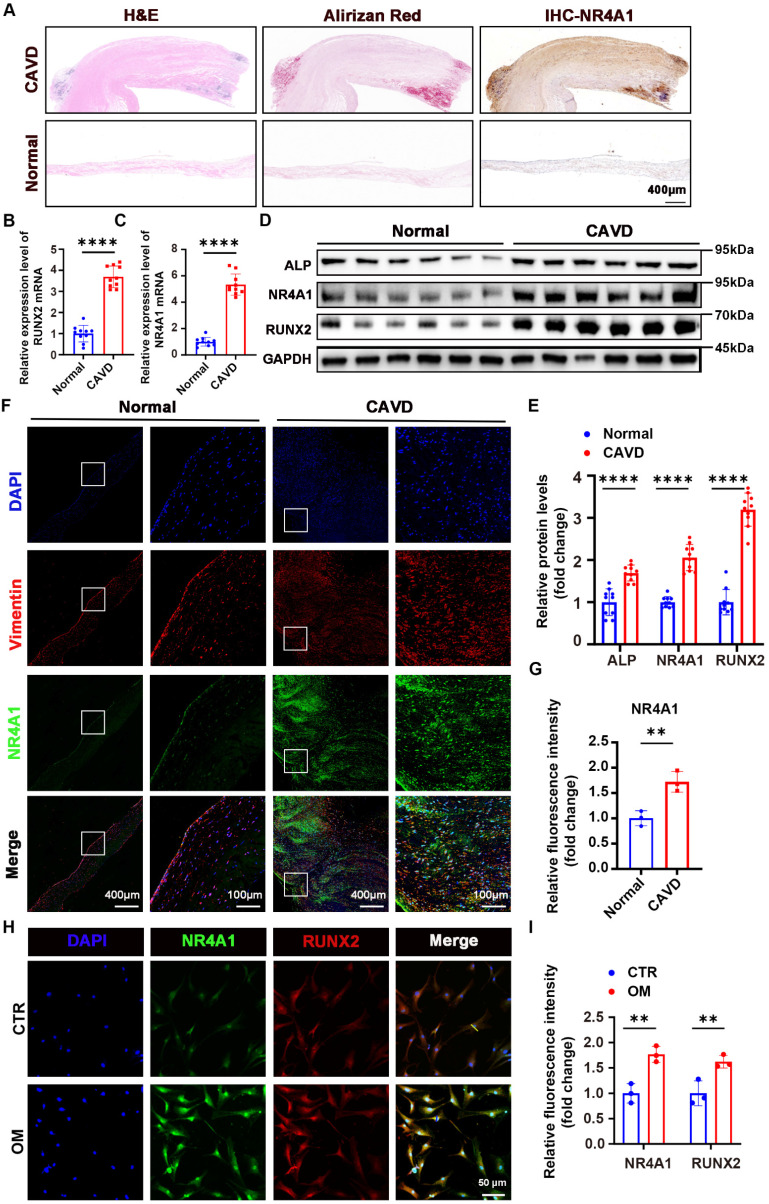
** NR4A1 expression is upregulated in VICs derived from human CAVs. (A)** Herein were shown representative images of aortic valve stained with hematoxylin and eosin (H&E) for general tissue profiling, Alizarin Red for calcium-related structures, and immunohistochemistry for NR4A1, scale bar: 400 μm. **(B-C)** qPCR analysis was performed to measure the mRNA levels of NR4A1 and RUNX2 in aortic valve tissues. Each group comprised 10 distinct human aortic valve specimens. **(D-E)** Western blotting validated the elevated levels of NR4A1, RUNX2, and ALP in calcified valves. Each group consisted of 10 separate human aortic valve tissue samples. **(F-G)** Immunofluorescence staining of NR4A1 and vimentin in aortic valve tissues (n = 3, each group), scale bar: 400 μm or 100 μm. **(H-I)** Immunofluorescence demonstrating co-localization of NR4A1 and RUNX2 in VICs under osteogenic stimulation (n = 3, each group), scale bar: 50 μm. The yellow lines and blue lines indicated the positions for fluorescence intensity measurement. All data are presented as means ± SD. Unpaired t-test was employed for statistical analysis. *p < 0.05, **p < 0.01, ***p < 0.001, ****p < 0.0001.

Aortic valves were collected to evaluate the differential expression of NR4A1 between the normal and CAVD groups. The origin of these aortic valves is described in the "Methods" section (**Supplementary Table S2**). Initially, the mRNA expression of NR4A1 was assessed in a set of 20 human aortic valve samples, which consisted of 10 samples from calcified aortic valves (CAVs) and 10 samples from normal controls. Hematoxylin-eosin staining, Alizarin Red staining, and immunohistochemical staining revealed a significant increase in calcium salt deposition and NR4A1 levels in the CAVD group. (**Figure 1A, Figure S2A, B**). PCR analysis of the tissues demonstrated an upregulation of NR4A1 mRNA in the CAVs group. (**Figure 1B, C**). Importantly, both immunohistochemical staining and immunoblotting demonstrated significantly higher NR4A1 expression in CAVs, with this upregulation of NR4A1 coinciding with elevated levels of osteogenic markers RUNX2 and ALP (**Figure 1A, D-E**). Furthermore, as the severity of CAVD increased, there was a significant elevation in both calcium deposition and NR4A1 levels (**Figure S2E-H**). These findings were further validated by immunofluorescence staining for NR4A1 and vimentin (**Figure 1F, G**), with vimentin serving as a marker for VICs. At the cellular level, a comparison of NR4A1 expression across different experimental conditions revealed significant colocalization of NR4A1 and RUNX2 in the nuclear region. What's more, a notable increase in NR4A1 expression was detected in the osteogenic media (OM) group (**Figure 1H, I, Figure S2C, D**).

### NR4A1 promotes osteogenic differentiation of VICs

**Figure 2:**
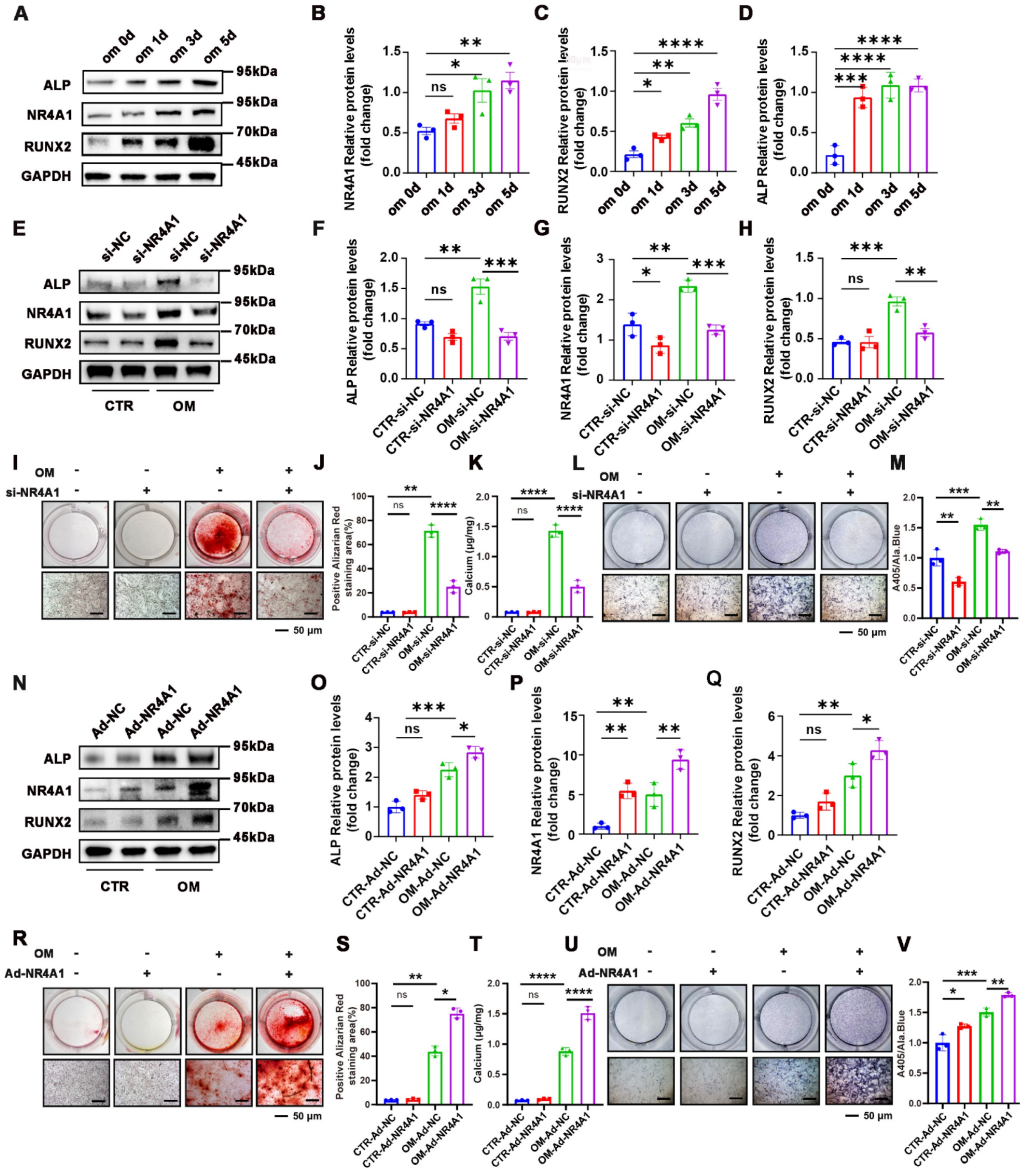
** NR4A1 Promotes Osteogenic Differentiation of VICs. (A-D)** WB experiments at time-point inductions (days 0, 1, 3, and 5 after the initiation of osteogenic culture) confirmed that the levels of NR4A1 gradually increased with the prolongation of OM induction time **(E-H)** Analysis of NR4A1, RUNX2, and ALP Expression in NR4A1-Knockdown VICs Using Western Blotting Technique. **(I-K)** Visual representation of the outcomes of Alizarin Red staining in VICs that were cultured in OM for 21 days subsequent to NR4A1 knockdown. **(L-M)** The visual display of ALP staining in VICs reveals the outcome after a 7-day culture in OM subsequent to NR4A1 knockdown. **(N-Q)** After infecting VICs with adenovirus carrying NR4A1 for 48 hours, the levels of osteogenic differentiation markers, namely ALP and RUNX2, were evaluated by WB and quantitatively analyzed. **(R-T)** Alizarin Red S staining of VICs after 21 days of osteogenic induction with NR4A1 overexpression. **(U-V)** Representative image of ALP staining of VICs after 7 days of osteogenic induction with NR4A1 overexpression (n= 3 per group). Scale bar: 50 μm. Data are shown as means ± SD. A rigorous statistical approach, involving ANOVA and Tukey's multiple comparisons test, was employed to assess significance. *p < 0.05, **p < 0.01, ***p < 0.001, and ****p < 0.0001.

We launched a series of subsequent experiments to explore whether NR4A1 exerts an effect on the osteogenic differentiation of VICs. NR4A1 expression increased during osteogenic induction, along with upregulation of osteogenic markers RUNX2 and ALP (**Figure 2A-D**), suggesting its involvement in VICs differentiation. To conduct a more in- depth assessment, VICs were treated with small interfering RNA (siNR4A1) for 8 hours or adenovirus (Ad-NR4A1) for 48 hours to silence or overexpress NR4A1. Western blot (WB) analysis revealed that silencing NR4A1 reduced ALP and RUNX2 expression (**Figure 2E-H**), while overexpression enhanced their levels (**Figure 2N-Q**). Similarly, Alizarin Red staining and ALP staining also demonstrated marked differences. Upon silencing of NR4A1, the activity of ALP and calcium salt deposition were significantly decreased (**Figure 2I - M**), while its overexpression resulted in an increase in these two aspects (**Figure 2R-V**).

### NR4A1 promotes proliferation of VICs

**Figure 3:**
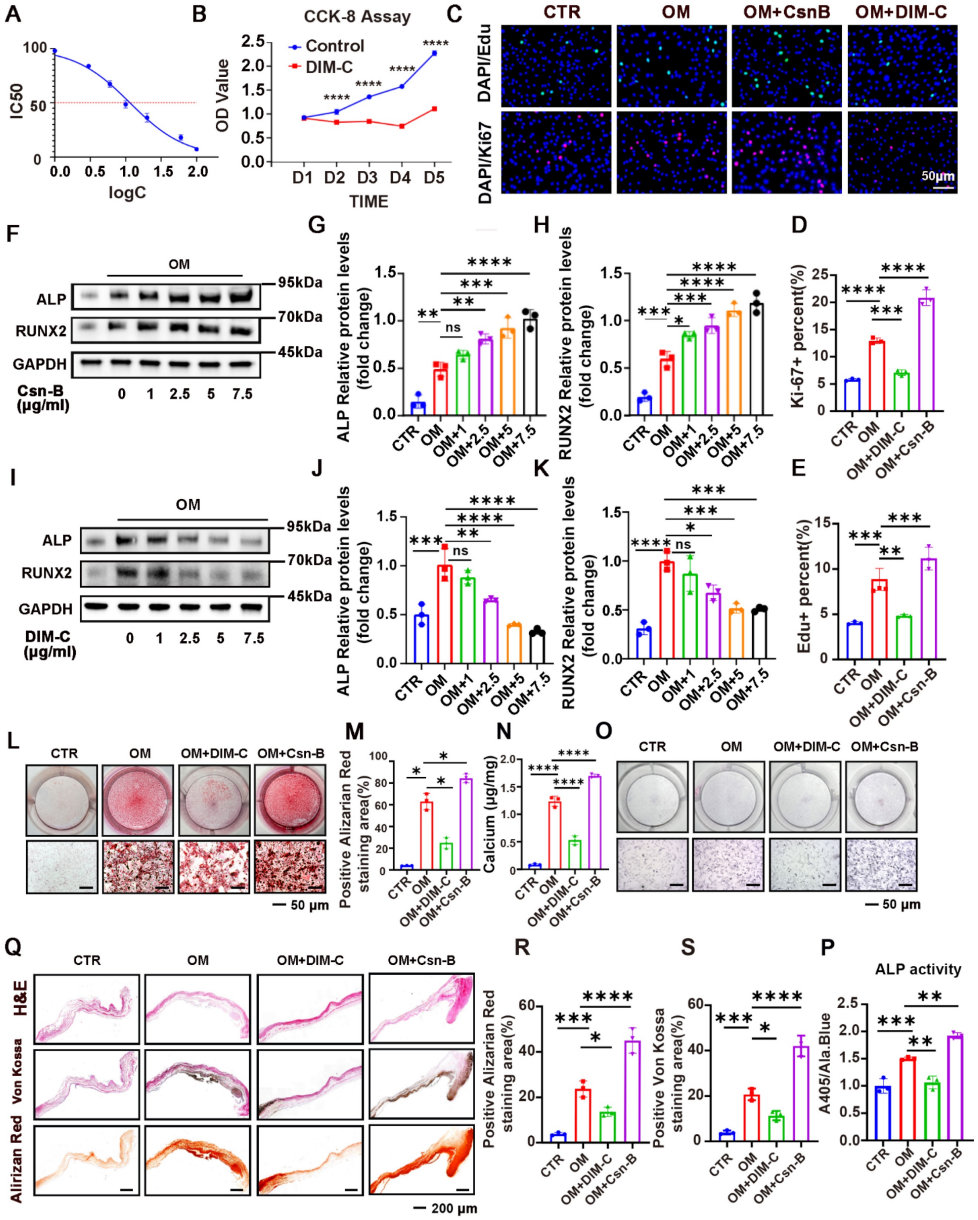
** Pharmacological activation or inhibition of NR4A1 using Csn-B or DIM-C modulates VICs proliferation and calcification. (A)** The 50% maximal inhibitory concentration (IC50) of DIM-C for VICs was ascertained, and the concentration was presented in logarithmic notation (log (c)). **(B)** The effect of DIM-C on the viability of VICs was evaluated using the CCK-8 assay. **(C-E)** In the context of VICs being cultured under osteogenic conditions with the addition of either Csn-B or DIM-C, Ki-67 and EdU staining was conducted. **(F-H, I-K)** Evaluation of protein levels in VICs through WB analysis after treatment with varying concentrations (0, 1, 2.5, 5, and 7.5 μg/ml) of Csn-B or DIM-C. **(L-P)** Visual representations of ALP and Alizarin Red staining resulted in VICs responding to 7.5 μg/ml of Csn-B or DIM-C treatment. **(Q-S)** The effects of Csn-B or DIM-C on aortic valves cultured in OM were evaluated through in-vitro tissue culture experiments, along with H&E, Von Kossa, and Alizarin Red staining. n = 3 per group. Scale bar: 50 μm or 200 μm. Data are presented as the mean ± SD. ANOVA with Tukey's multiple comparisons test. * p < 0.05, ** p < 0.01, *** p < 0.001, and **** p < 0.0001.

The IC50 value was calculated to evaluate the cytotoxicity of DIM-C on VICs, revealing significant toxic effects at concentrations exceeding 10 μg/mL (**Figure 3A**). Measuring the Proliferative Response of VICs to Csn-B (an NR4A1 agonist) and DIM-C (an NR4A1 inhibitor) with CCK-8 Assays, Ki - 67 Staining, and EdU Staining. The experimental results revealed that DIM - C, an inhibitor of NR4A1, led to an almost complete halt in the growth of VICs (**Figure 3B**). By means of Ki-67 and EdU staining, it was further verified that DIM-C significantly depressed VICs proliferation, and conversely, Csn-B notably stimulated VICs proliferation. (**Figure 3C-E**). Western blot analysis further corroborated these findings, demonstrating that RUNX2 and ALP expression exhibited a concentration-dependent increase under Csn-B treatment (**Figure 3F-H**), whereas the opposite trend was observed with DIM-C treatment (**Figure 3I-K**). Additionally, Csn-B enhanced calcium deposition and ALP activity in VICs induced by OM, while DIM-C exerted the opposite effects (**Figure 3L-P**). Through in-vitro aortic valve tissue culture experiments, along with von Kossa staining and Alizarin Red staining, the results demonstrated that Csn-B could significantly augment calcium salt deposition, while DIM-C could markedly reduce it. (**Figure 3Q-S**). Additionally, WB analysis showed that after 7 days of osteogenic medium (OM) treatment, followed by 7 days of DIM-C intervention under OM conditions, there was a significant reduction in the calcification markers ALP and RUNX2 (**Figure S2I-K**). Alizarin Red staining further demonstrated that after 21 days of OM induction, the subsequent 21-day DIM-C intervention under OM conditions markedly decreased calcium deposition (**Figure S2L-N**). These results collectively indicated that, *in vitro*, activation of NR4A1 promotes VICs proliferation and exacerbates aortic valve calcification.

### Determining NR4A1 candidate target genes crucial for osteogenic differentiation

**Figure 4:**
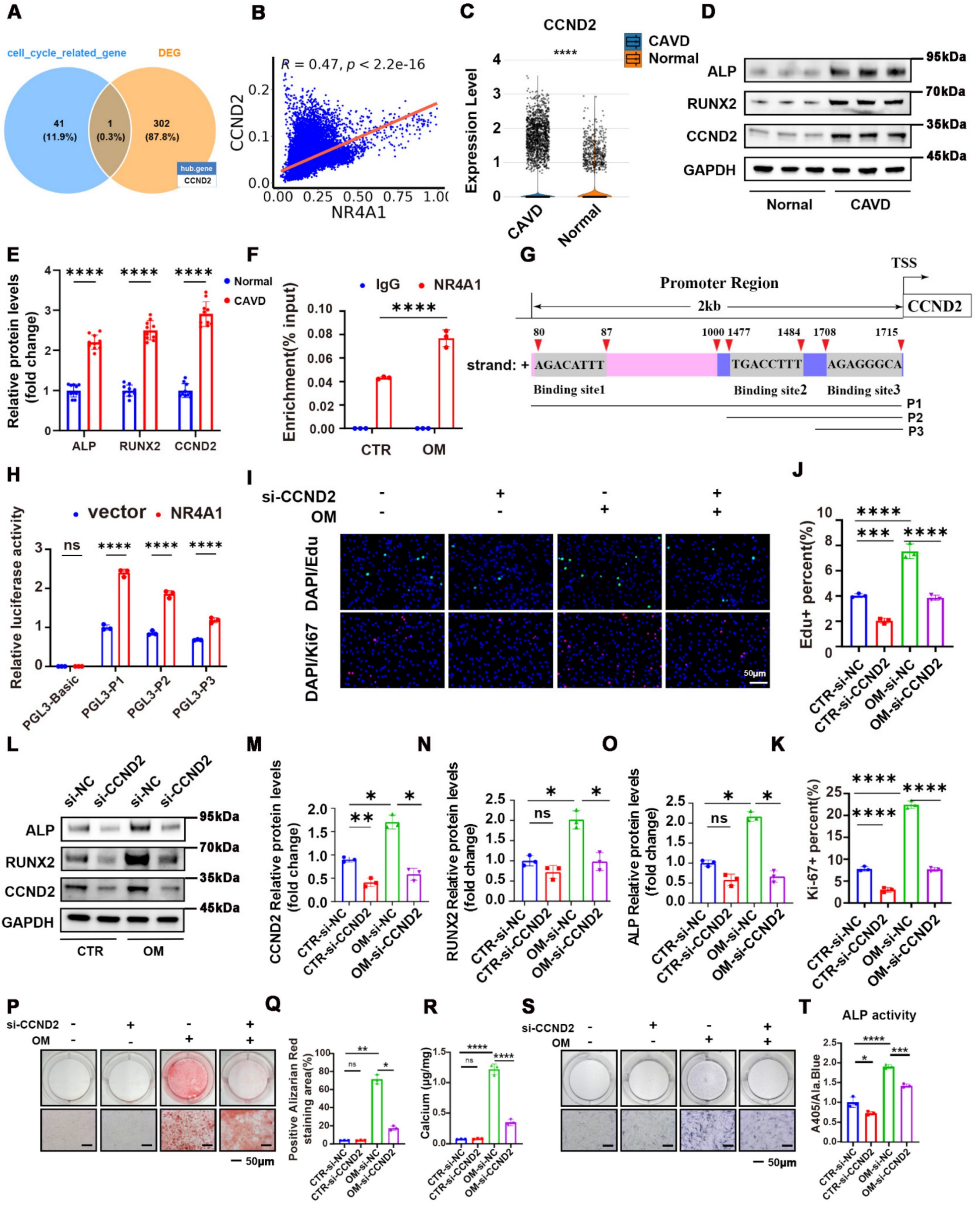
** CCND2, a target gene regulated by NR4A1, is capable of promoting the osteogenic differentiation of VICs. (A)** Venn diagram illustrating the overlap between differentially expressed genes and genes associated with cell cycle regulation. **(B)** Scatter plot showing the correlation between NR4A1 and CCND2 expression levels in single cells. **(C)** Violin plot illustrating CCND2 expression levels in calcified valves versus normal valves. **(D-E)** In the differential valve tissues, the levels of CCND2, as well as the calcification markers ALP and RUNX2, were assessed and quantified by WB analysis (n = 10, each group). **(F)** Chromatin immunoprecipitation coupled with quantitative PCR (ChIP-qPCR) assay showing the enrichment of the CCND2 promoter region by NR4A1 in VICs under CTR and OM conditions (n = 3, each group). **(G)** Schematic illustration of the CCND2 gene promoter region (2 kb upstream of the transcription start site, TSS). The locations of three putative NR4A1 binding sites (Binding site1, Binding site2, Binding site3), predicted by Jaspar, are indicated, along with their respective DNA sequences. P1, P2, and P3 represent different promoter fragment plasmids. **(H)** Luciferase reporter assay showing relative luciferase activity in VICs transfected with the empty vector control (PGL3-Basic, which serves as a negative control with no luciferase expression) or different truncated CCND2 promoter plasmids (PGL3-P1, PGL3-P2, PGL3-P3), either with empty vector (blue) or NR4A1-overexpressing vector (red) (n = 3, each group). **(I-K)** Ki-67 and EdU staining of VICs under osteogenic stimulation following CCND2 knockout. **(L-O)** WB experiments of CCND2, RUNX2, and ALP expression in VICs following CCND2 silencing (n = 3, each group). **(P-T)** Following the silencing of the CCND2 gene, representative images were obtained from Alizarin Red staining and ALP staining in VICs. (n = 3, each group). Scale bar: 50 μm. Data are means ± SD. *p < 0.05, **p < 0.01, ***p < 0.001, ****p < 0.0001(ANOVA with Tukey's multiple comparisons test).

To explore the target genes through which NR4A1 induced VICs calcification, we conducted transcriptomic sequencing on samples treated with OM+si-NC and OM + siNR4A1 (3:5). We intersected the differentially expressed genes with genes associated with cell cycle regulation (**Supplementary Table S3**) to identify potential overlapping candidates. The Venn diagram suggests that NR4A1 may regulate the cell cycle of VICs through the modulation of CCND2. (**Figure 4A**). The heatmap visualizes the expression patterns of selected differentially expressed genes between the two groups, while Western blot analysis confirmed that NR4A1 knockout significantly downregulates the expression level of the cyclin CCND2 (**Figure S3A-D**). Meanwhile, single-cell analysis showed upregulation of CCND2 in CAVs, with a correlation of 0.47 between CCND2 and NR4A1 (**Figure 4B, C**). In addition, immunoblot analysis presented data indicating that the expression levels of CCND2, as well as the calcification markers ALP and RUNX2, were greater in calcific valvular tissue as opposed to normal aortic valves (**Figure 4D, E**). Additionally, chromatin immunoprecipitation (ChIP) analysis verified the binding of NR4A1 to the CCND2 promoter, with osteogenic stimulation further potentiating this binding event (**Figure 4F**). The Jaspar database was utilized to predict potential NR4A1-binding sites in the CCND2 promoter region, and a panel of luciferase reporter plasmids (P1, P2, P3) containing truncated fragments of the CCND2 promoter was generated to assess NR4A1's impact on luciferase activity (**Figure 4G**). Our data revealed that overexpression of NR4A1 led to a significant elevation in the luciferase activity of these constructs (**Figure 4H**). Furthermore, the results of Ki-67 and EdU staining showed that knockout of CCND2 significantly inhibits cell proliferation. (**Figure 4I, J**). Moreover, the reduction in CCND2 expression was associated with the inhibition of RUNX2 and ALP expression in VICs induced by OM (**Figure 4L-K**). Furthermore, the outcomes from ALP activity assays, in combination with the results of Alizarin Red staining, indicated that downregulation of CCND2 alleviated ALP activity and reduced the degree of OM - induced calcification in VICs (**Figure 4P-T**). These discoveries further authenticate the function of CCND2 during the course of valvular calcification.

### NR4A1 promotes VICs proliferation and calcification via upregulation of CCND2

**Figure 5:**
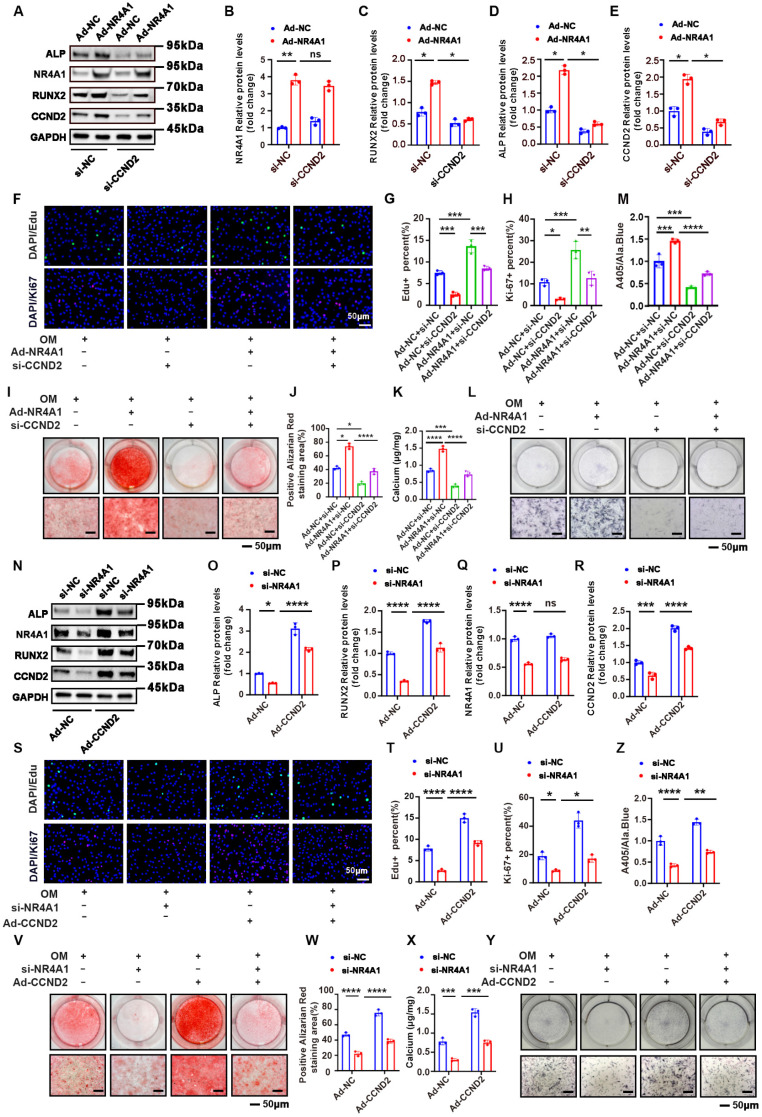
** Silencing of CCND2 mitigates the osteogenic differentiation phenotype induced by NR4A1 overexpression in VICs. (A-E)** Knockdown of CCND2 was able to inhibit the elevation of the levels of calcification markers ALP and RUNX2 induced by overexpression of NR4A1, as demonstrated by WB (n = 3, each group). **(F-H)** Visual representations of Ki-67 and EdU staining in VICs when NR4A1 was overexpressed and CCND2 was silenced (n = 3, each group). **(I-M)** Determination of calcified nodules and ALP activity in valvular interstitial cells with CCND2 gene silencing and NR4A1 overexpression (n = 3, per group). **(N-R)** Western blot analysis of calcification markers ALP and RUNX2 in VICs with NR4A1 silencing and CCND2 overexpression. **(S-U)** Visual representations of Ki-67 and EdU staining in VICs with NR4A1 silencing and CCND2 overexpression. **(V-Z)** Determination of calcified nodules and ALP activity in valvular interstitial cells with NR4A1 silencing and CCND2 overexpression. (n = 3, per group). Scale bar: 50 μm. Data are means ± SD. One-way ANOVA (*p < 0.05, **p < 0.01, ***p < 0.001, ****p < 0.0001)

Subsequently, we conducted a rescue experiment to determine whether NR4A1 exerts its effects via upregulation of CCND2. Immunoblotting revealed that CCND2 knockout effectively attenuated the NR4A1-induced upregulation of RUNX2 and ALP (**Figure 5A-E**). Moreover, CCND2 depletion suppressed the cell proliferation triggered by NR4A1 overexpression (**Figure 5F-H**). Additionally, ALP activity assays and Alizarin Red staining corroborated these findings, showing that CCND2 knockout reduced both ALP activity and calcium deposition (**Figure 5I-M**). In contrast, the calcification effects induced by NR4A1 knockdown were reversed by the overexpression of CCND2 (**Figure 5N-Z**). Taken together, these results suggested that NR4A1 promoted cell proliferation and accelerated cell osteogenic transformation by upregulating CCND2 in human VICs.

### Determining NR4A1 as a gene targeted by JMJD3-H3K27me3 in response to osteogenic stimulation

**Figure 6:**
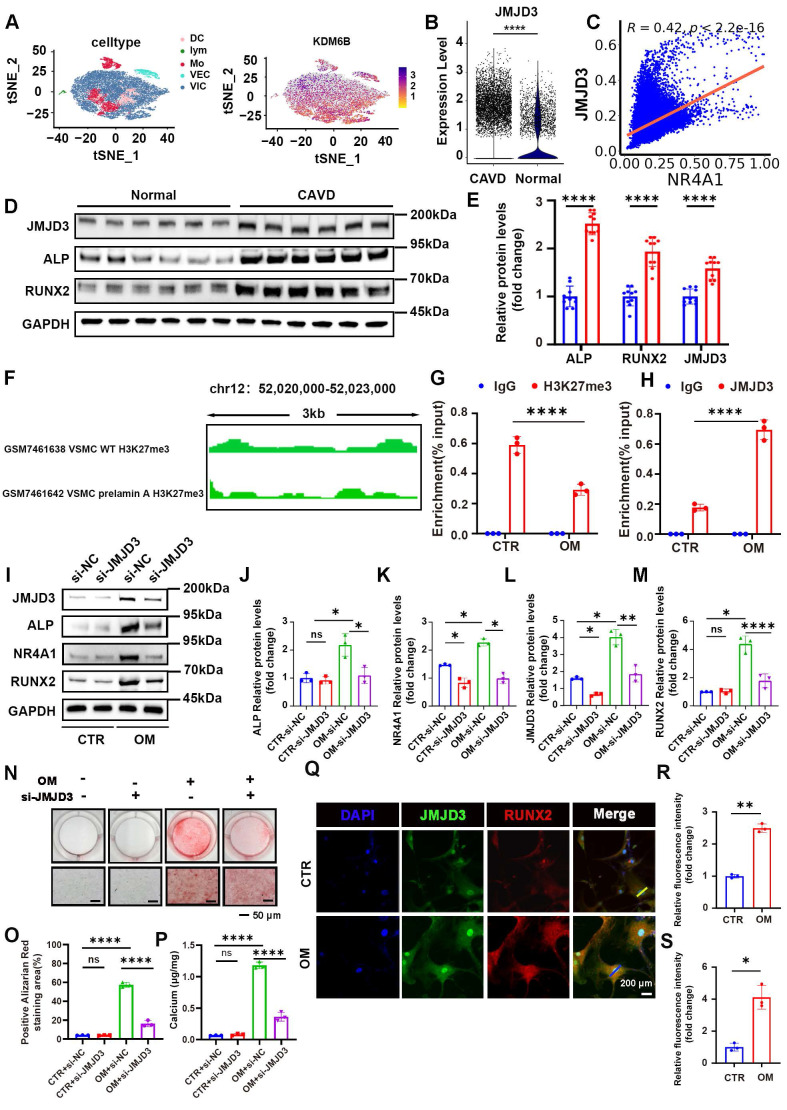
** JMJD3 upregulates NR4A1 expression in VICs following osteogenic stimulation. (A)** The left-hand panel showed a t-distributed stochastic neighbor embedding (t-SNE) plot that displays cell types, while the right-hand panel presented the distribution of the JMJD3 gene within the cell clustering (t - SNE) plot. **(B)** A violin plot was utilized to illustrate the differential expression of JMJD3 between normal and calcified valves. **(C)** A scatter plot was employed to demonstrate the correlation between JMJD3 and NR4A1. **(D, E)** WB analysis ofJMJD3, RUNX2, and ALP expression in the aortic valve (n = 10, each group). **(F)** IGV tracks depict H3K27me3 enrichment at the chromosomal region chr12: 52,020,000-52,023,000 (spanning 3 kb) in vascular smooth muscle cells (VSMCs) for ChIP-Seq profiles related to vascular calcification. **(G-H)** ChIP-qPCR assay showing the enrichment of H3K27me3 and JMJD3 at the NR4A1 locus in VICs under CTR and OM conditions. IgG was used as a negative control in both assays (n = 3, each group). **(I-M)** After knocking out JMJD3, the alterations in the protein levels of NR4A1 as well as the calcification markers ALP and RUNX2 were detected by WB (n = 3, each group). **(N-P)** The Alizarin Red staining images were presented for samples with either JMJD3 gene knockdown or without JMJD3 gene knockdown (n = 3, each group). Scale bar: 50 μm.**(Q-S)** Immunofluorescence examination revealed a marked upregulation of JMJD3 expression under osteogenic stimulation (n = 3, each group). The yellow lines and blue lines indicated the positions for fluorescence intensity measurement. Scale bar: 20 μm. Data are means ± SD. One-way ANOVA, followed by Tukey's multiple comparisons test, was employed to analyze the data. * p < 0.05, ** p < 0.01, *** p < 0.001, and **** p < 0.0001.

After confirming the indispensable function of NR4A1 in the course of VICs' osteogenic differentiation, we further explored the upstream histone demethylases responsible for regulating NR4A1 expression. To this end, single-cell analysis of 11 common histone demethylases was performed, revealing significant differences between normal and CAVs, along with their correlation to NR4A1 expression (**Figure S4A-K**). The top four demethylases with the strongest correlation coefficients were selected for experimental validation (**Figure S5A-L**). Notably, the inhibition or silencing of JMJD3 engendered a marked diminution in NR4A1 levels and effectively reversed the osteogenic differentiation induced by osteogenic stimulation (**Figure 6I-M**). The t-SNE plot illustrated the distribution of JMJD3 expression across distinct cellular subpopulations (**Figure 6A**). Meanwhile, single-cell analysis showed upregulation of JMJD3 in CAVs, with a correlation of 0.42 between JMJD3 and NR4A1 (**Figure 6B, C**). Furthermore, immunoblot analysis revealed elevated expression levels of ALP, RUNX2, and JMJD3 in calcified valvular tissue compared to normal aortic valves (**Figure 6D, E**). In cultured human VICs, osteogenic stimulation led to a time-dependent increase in JMJD3 protein levels, while H3K27me3 levels decreased in a time-dependent manner (**Figure S6A-E**). Given the mechanistic parallels between vascular calcification and valvular calcification, we analyzed H3K27me3 ChIP-seq datasets derived from vascular calcification models to evaluate the enrichment signal of H3K27me3 at the NR4A1 promoter region. Notably, the H3K27me3 signal intensity at the NR4A1 promoter was significantly diminished in the calcification group compared to the normal control group (**Figure 6F**). To investigate the dynamic changes of histone modifications during VICs osteogenic differentiation, we performed chromatin immunoprecipitation coupled with quantitative PCR (ChIP-qPCR) using antibodies against H3K27me3 and JMJD3 in VICs under CTR and OM conditions. ChIP-qPCR revealed a substantial reduction in H3K27me3 enrichment at the target locus in OM-treated VICs compared to CTR (**Figure 6G**), whereas it demonstrated a marked increase in JMJD3 occupancy at the same locus in the OM group relative to CTR (**Figure 6H**). The results of Alizarin Red staining demonstrated that the downregulation of JMJD3 attenuates the degree of calcification induced by osteogenic differentiation (OM) (**Figure 6N-P**). At the cellular level, JMJD3 expression was compared across various conditions, showing significant colocalization of JMJD3 with RUNX2 in the nucleus, and an upward trend in NR4A1 expression in the osteogenic media (OM) group (**Figure 6Q-S, Figure S6I, J**). An in-vitro culture model of valve tissue was utilized by us to examine the impact of GSK-J1 on calcium salt deposition. To assess the impact on calcium deposition, H&E, Von Kossa, and Alizarin Red staining were performed, revealing that GSK-J1 effectively lessened calcium deposition within the valve tissue (**Figure S6F, H**).

### Inhibition of NR4A1 alleviates aortic valve calcification *in vivo*

**Figure 7:**
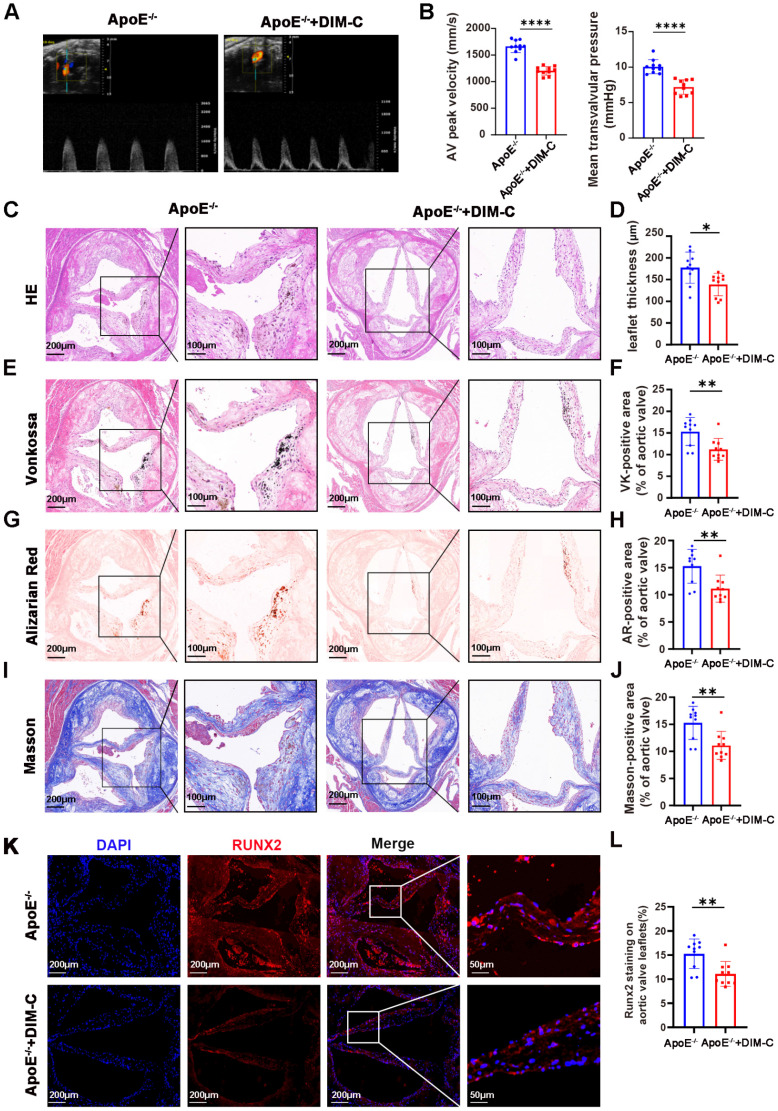
** Suppression of NR4A1 *in vivo* mitigates aortic valve calcification. (A-B)** Echocardiographic assessment of maximal transvalvular flow velocity and mean transvalvular pressure gradient in ApoE^-/-^ mice and ApoE^-/-^ mice treated with DIM-C (n = 10 per group). **(C-J)** Histological analyses of aortic valves from ApoE^-/-^ mice and ApoE^-/-^ + DIM-C mice, including staining with H&E (C-D), Von Kossa (E-F), Alizarin Red (G-H), and Masson's Trichrome (I-J). Scale bar: 200 μm or 100 μm. **(K-L)** Immunofluorescence staining for RUNX2 (red) in aortic valves (nuclear counterstaining with DAPI, blue) from ApoE^-/-^ mice and ApoE^-/-^ + DIM-C mice. Scale bar: 200 μm or 50 μm. Values are mean ± SD. All statistical analyses were carried out using a unpaired Student's t-test. * p < 0.05, ** p < 0.01, *** p < 0.001, and **** p < 0.0001.

To translate our *in vitro* observation that NR4A1 promotes the osteogenic differentiation of valvular interstitial cells (VICs) into an *in vivo* context, we sought to elucidate the impact of NR4A1 inhibition on aortic valve calcification. Both groups of ApoE^-/-^ mice were subjected to a 24-week high-fat diet, with one group receiving DIM-C treatment and the other group not receiving DIM-C. Echocardiographic assessments revealed that, in stark contrast to the ApoE^-/-^ group without DIM-C treatment, mice in the ApoE^-/-^ + DIM-C cohort exhibited a marked reduction in both the maximal transvalvular jet flow velocity and the mean transvalvular pressure gradient (**Figure 7A-B**).

A comprehensive comparison of echocardiographic and hemodynamic parameters in ApoE^-/-^ mice is detailed in **Supplementary Table S4**, which confirmed that the observed effects were specific to valvular pathology rather than global cardiac dysfunction. Histological examinations, employing H&E, Von Kossa, Alizarin Red, and Masson staining, further substantiated our findings. Relative to the ApoE^-/-^ group without DIM-C intervention, DIM-C treatment elicited a significant attenuation of aortic valve thickening in ApoE^-/-^ mice (**Figure 7C-D**). Concomitantly, Von Kossa and Alizarin Red staining unveiled a pronounced reduction in calcium deposition within the aortic valve leaflets in the ApoE^-/-^ + DIM-C group, while Masson staining demonstrated a decrease in collagen formation (**Figure 7E-J**). Immunofluorescence staining for RUNX2, a pivotal osteogenic marker, revealed a striking downregulation of RUNX2 expression in the ApoE^-/-^ + DIM-C group when compared to the ApoE^-/-^ group (**Figure 7K-L**). Furthermore, to assess the potential side effects of DIM-C, we collected samples from major organs and blood for analysis. As shown in **Figure S7A-D**, no significant differences were observed in the biochemical parameters related to liver and kidney function, including alanine aminotransferase (ALT), aspartate aminotransferase (AST), creatinine (CREA), and uric acid (UA), between the ApoE^-/-^ and ApoE^-/-^+ DIM-C groups. Additionally, HE staining revealed no significant effects of DIM-C on the heart, liver, spleen, lungs, or kidneys (**Figure S7E**). Collectively, these *in vivo* results underscore that inhibition of NR4A1 can potently mitigate aortic valve calcification, as evidenced by improvements in hemodynamic parameters, reductions in histological hallmarks of calcification and fibrosis, and diminished expression of a critical osteogenic marker.

## Discussion

CAVD has become much more prevalent, mainly due to the aging of the global population 21. Although several risk factors for aortic valve calcification have been identified, the underlying molecular mechanisms remain poorly understood 22. Currently, valve repair or replacement remains the only effective clinical treatment option for patients with CAVD 21. NR4A1, an orphan nuclear receptor belonging to the NR4A subfamily of nuclear hormone receptors 23, has emerged as a pivotal regulator in the pathophysiology of various cardiovascular maladies, among which are atherosclerosis, vascular calcification, and cardiac remodeling 5-9. This study uncovers novel evidence indicating that NR4A1 facilitates the calcification of VICs by promoting cellular proliferation and enhancing osteogenic differentiation, both through the modulation of CCND2. Furthermore, we observed that JMJD3 upregulates NR4A1 expression during the calcification process in VICs.

Despite the fact that the function of NR4A1 within the realm of cardiovascular biology has been explored comprehensively, its functional implications in cardiovascular pathology remain a subject of considerable controversy and debate. Previous studies have indicated that NR4A1 may attenuate atherosclerosis 5; conversely, an increasing body of literature suggests that NR4A1 may promote the progression of atherosclerosis and vascular calcification 6-8. Notably, knockdown of NR4A1 has been demonstrated to ameliorate mitochondrial dysfunction and dysregulation of glucose and lipid metabolism in cardiac tissues, thereby attenuating myocardial microvascular ischemia-reperfusion injury 9. Hence, we hypothesize that the dual role of NR4A1 in cardiovascular diseases may be attributable to cell-type-specific effects or variations in experimental conditions. Our research reveals that the overexpression of NR4A1 notably boosts the levels of osteogenic markers, such as RUNX2 and ALP, and also facilitates the accumulation of calcium within VICs cultured in OM. On the contrary, the suppression of NR4A1 diminishes the osteogenic differentiation capacity of VICs. Collectively, these observations lead to the inference that NR4A1 acts as a promoter of calcification in VICs.

Mounting evidence increasingly supports cellular proliferation as a central mechanism driving the pathogenesis and progression of various cardiovascular diseases. Atherosclerosis, in particular, is a multifactorial disorder characterized by persistent inflammation and uncontrolled cellular proliferation, both of which contribute significantly to the disease's development and advancement 12. Endothelial cell (EC) dysfunction and aberrant proliferation of vascular smooth muscle cells (VSMCs) significantly contribute to the occurrence and development of atherosclerosis and vascular calcification 10, 11. The progression of calcific aortic valve disease (CAVD) closely resembles ectopic ossification. In the early stages of aortic valve stenosis, the proliferation and osteogenic differentiation of VICs emerge as critical phenotypic events driving disease development. Upon activation, VICs undergo excessive proliferation and produce an overabundance of extracellular matrix (ECM) proteins, which not only promote fibrosis within the valve tissue but also accelerate osteogenic differentiation, leading to the transformation of VICs into bone-like tissue. This cascade of events ultimately results in valve calcification and stenosis 15. Furthermore, Huang et al. have elucidated that Gli1 promotes the proliferation of VICs, thereby accelerating valvular calcification 24. Additionally, Huang et al. identified that andrographolide inhibits cell cycle-related proteins CDK1 and E2F1, thereby suppressing cellular proliferation and subsequently mitigating valvular calcification 25. At the same time, Yao et al. demonstrated that cyclin D1 plays a pivotal role in the fibrotic response of valve interstitial cells, with inhibition of cyclin D1 effectively abolishing the fibrosis induced by Neurotrophin 3 15.

It is noteworthy that several classical signaling pathways play key roles in the progression of CAVD by regulating cell proliferation and differentiation. Studies have shown that BMP4 plays a critical role in the osteogenic differentiation of VICs, and the use of BMP4 antagonist NOG can effectively reduce the osteogenic-like phenotype of VICs characterized by overexpression of SMA, FN, OPN, and ALP 26. Moreover, β-catenin, as the core mediator of the Wnt signaling pathway, also promotes stenosis and calcification in aortic valve stenosis. In both hypercholesterolemic mouse models and human AVS patients, BMP signaling and Wnt/β-catenin signaling are significantly and persistently co-activated 27. Further mechanistic studies revealed that the activation of Wnt signaling is synergistically upregulated with the expression of CCND2 and Bmp4. Both of their promoter regions contain TCF/LEF and Smad binding sites, and Lef1 with pSmad1/5/8 can form a “triple complex” to co-activate the transcription of these genes 28. As a key ligand of the BMP pathway, Bmp4 high expression in VICs can regulate the synthesis and degradation of ECM, promoting the synthesis of osteopontin, collagen, and other components.

Based on the aforementioned evidence, it is speculated that the CCND2 overexpression observed in this study may exacerbate ECM remodeling through synergistic action with Bmp4. Specifically, the over-proliferating VICs themselves secrete more ECM components, and the overexpression of CCND2 may further enhance the activity of the Bmp4-Smad signaling pathway, driving excessive ECM deposition or abnormal ECM composition (such as collagen imbalance). In the long term, this could lead to valve fibrosis and stiffness, impairing the normal diastolic and systolic functions of the valve, thus laying the groundwork for valve stenosis. Additionally, the AKT signaling pathway plays an important role in the development of CAVD 24, 29. The activation of AKT can promote the proliferation of VICs and collagen deposition, thereby driving the fibrosis and calcification of the valve. Similar to its role in atherosclerosis, AKT regulates the progression of the disease by modulating cell proliferation, migration, and inflammation. Research has shown that AKT inhibitors, such as MK2206, reduce lipid deposition, inhibit VSMCs proliferation and migration, and attenuate inflammation, thus slowing the disease progression 30. Mechanistically, AKT signaling promotes VICs proliferation through the indirect activation of cyclin D1 15. Both cyclin D1 and cyclin D2 regulate the transition from G1 to S phase of the cell cycle, thereby accelerating cell proliferation 31, 32. Studies have shown that cyclin D1 activation accelerates VICs proliferation, leading to greater accumulation of ECM proteins, which contributes to valve sclerosis. In this study, we demonstrate a direct mechanism in which NR4A1 binds to the cyclin D2 promoter, thereby promoting its expression and driving VICs proliferation. Taken together, our findings suggest that the JMJD3-NR4A1-CCND2 axis potentially facilitates CAVD, analogous to the canonical AKT-cyclin D1 pathway. In addition, this axis may intersect with established CAVD regulatory pathways, such as BMP, Wnt, and AKT, collectively contributing to the onset and progression of valve sclerosis. Future research should further explore the interactions and synergistic effects between these pathways to enhance our understanding of CAVD pathogenesis.

The methylation status of histones exerts a profound and indispensable influence on the regulation of gene expression 33. Histone demethylases, by removing these methyl modifications, typically alter chromatin structure, thereby influencing transcriptional activity 34. Numerous studies have confirmed that both KDM6A and JMJD3 are capable of propelling the advancement of osteogenic differentiation in mesenchymal stem cells (MSCs) and cranial bone cells 35, 36. Specifically, JMJD3 has been shown to upregulate the expression of BMAL1, thereby enhancing osteogenic differentiation of MSCs 37. Although existing research has demonstrated that JMJD3 can promote the progression of osteoarthritis by upregulating NR4A1 expression, its function within the context of CAVD continues to be an enigma 38. Through experimental validation, we have demonstrated that silencing JMJD3 downregulates NR4A1, consequently reducing the expression of osteogenic markers, among which are RUNX2 and ALP.

In summary, our study reveals a significant upregulation of NR4A1 in CAVD. We further demonstrate that NR4A1 plays a crucial role in the proliferation and extracellular matrix synthesis of VICs by upregulating the expression of the cell cycle-related protein CCND2. Notably, we also identify that the osteogenic induction of NR4A1 is mediated by JMJD3. Silencing JMJD3 not only reduces NR4A1 expression but also effectively inhibits the osteogenic differentiation of VICs.

This study has several limitations that should be acknowledged. Firstly, in verifying the role of NR4A1 in CAVD *in vivo*, we only used an NR4A1 inhibitor rather than NR4A1 knockout mice. Thus, in subsequent studies, we plan to establish NR4A1^-/-^ApoE^-/-^ double-knockout mice, which will avoid potential off-target effects of small-molecule inhibitors and enable better observation of its *in vivo* impact on aortic valve calcification. Secondly, although we demonstrated the regulatory relationship between NR4A1, JMJD3, and CCND2 in VIC osteogenic differentiation *in vitro*, we have not yet verified the impacts of JMJD3 and CCND2 on this process *in vivo*. In the future, we will use adeno-associated viruses combined with double-knockout mice to verify this process. Finally, our current research has focused exclusively on the function of VICs during CAVD progression. However, CAVD pathogenesis is a complex process involving multiple cell types, and valvular endothelial cells (VECs)—as the outermost barrier of the aortic valve—play a critical role in maintaining valvular homeostasis (e.g., regulating vascular permeability and inhibiting inflammatory cell infiltration). Dysfunction of valvular endothelial cells (VECs), such as impaired nitric oxide (NO) biosynthesis, may initiate or exacerbate valvular interstitial cell (VIC)-mediated calcification 39, 40. In future work, we will further explore VEC-VIC cell-cell interactions (e.g., through VEC-VIC co-culture models) to clarify how VEC dysfunction modulates VIC osteogenic differentiation. Integrating research on both cell types will provide a more comprehensive understanding of CAVD pathogenic mechanisms and lay a foundation for developing multi-target therapeutic strategies.

## Supplementary Material

Supplementary figures.

Supplementary tables.

## Data Availability

The authors affirm that the data underpinning the findings of this research can be provided upon request.

## Abbreviations

CAVD: calcific aortic valve disease
NR4A1: nuclear receptor subfamily 4 group a member 1
VICs: valve interstitial cells
RUNX2: runt-related transcription factor 2
OM: osteogenic medium
VSMCs: vascular smooth muscle cells
VECs: valvular endothelial cells
JMJD3: jumonji domain containing 3
CCND2: cyclin D2
