# Supplementary material for: Orphan Nuclear Receptor NR4A1 Promotes Proliferation and Osteogenic Differentiation of Valvular Interstitial Cells through Activation of CCND2
Source: Int J Biol Sci. 2026 Feb 26;22(6):3174–90. doi: 10.7150/ijbs.122863 (PMC13050472; doi:10.7150/ijbs.122863)
Supplement: Supplementary file 1 — Supplementary figures. [file ijbsv22p3174s1.pdf]

-

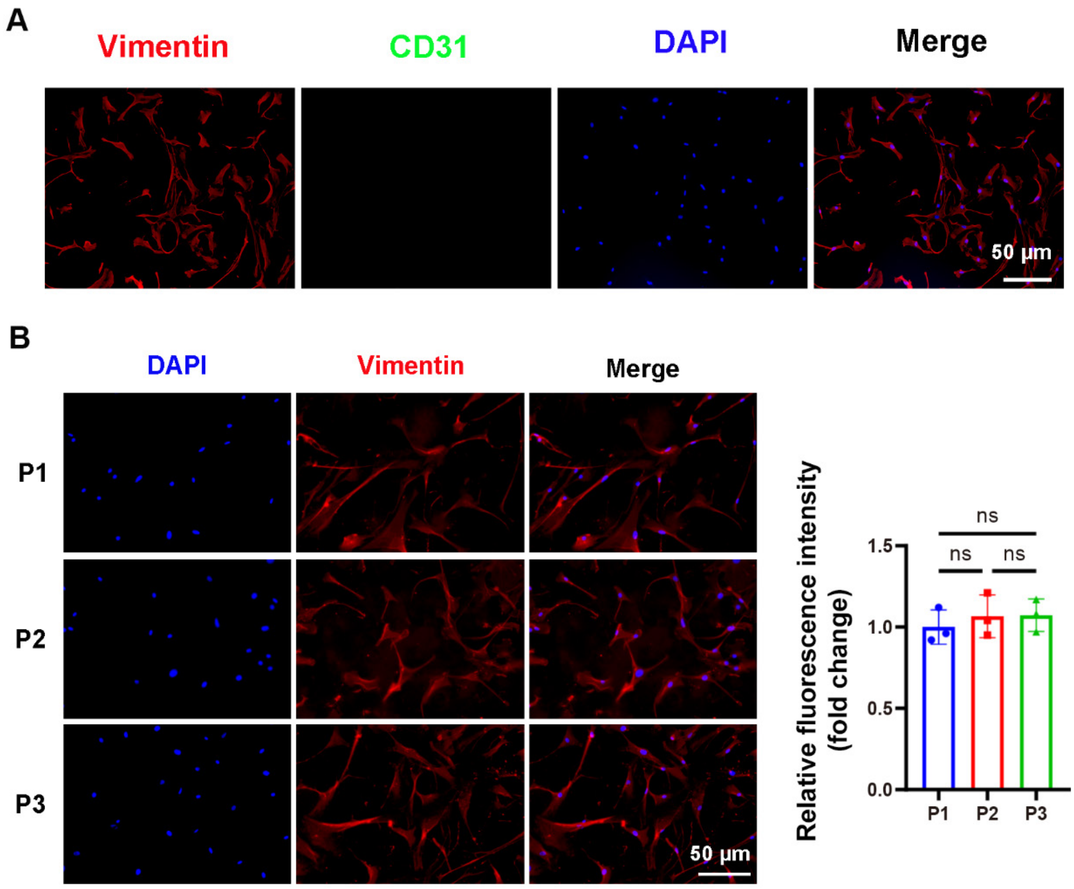

Figure S1. (A) Immunofluorescence staining of vimentin (red) and CD31 (green) in VICs. DAPI was used for nuclear counterstaining (blue). (B) Immunofluorescence staining showing the expression of Vimentin (red) in VICs at each passage (P1-P3). DAPI was used for nuclear counterstaining (blue). Scale bar: 50 μm.

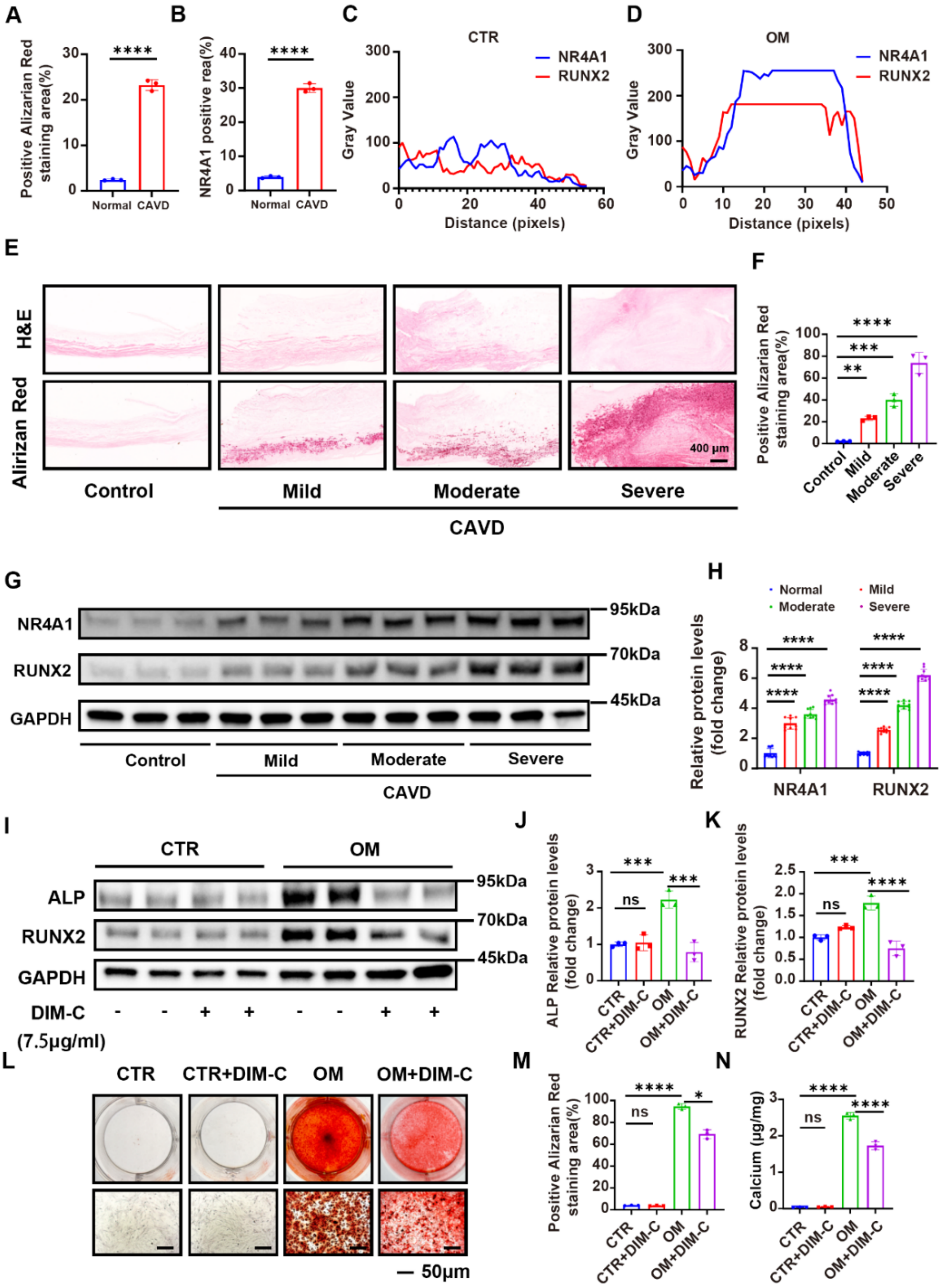

Figure S2. (A, B) Quantitative analysis of the positive areas in alizarin red staining and NR4A1 immunohistochemical staining in normal and calcified valve tissues. (C, D) Colocalization analysis of NR4A1 and RUNX2. Fluorescence intensity of NR4A1 and RUNX2 was measured at the positions indicated by the yellow and blue lines. (E, F) Representative images of Alizarin Red S staining (red) for detection of calcified nodules in aortic valve tissues. Scale bar: 400 μm. (G, H) Representative images of western blots for NR4A1 and Runx2 protein expression in aortic valve tissues. (n = 10, each group) (I-K) WB analysis of ALP and RUNX2 levels after 7 days of OM induction, followed by 7 days of DIM-C intervention under OM conditions, to assess the effect of DIM-C on calcification markers (n = 3, each group). (L-N) Representative alizarin red staining

after 21 days of OM induction, followed by 21 days of DIM-C intervention under OM conditions (n = 3, each group). Scale bar: 50 μm.

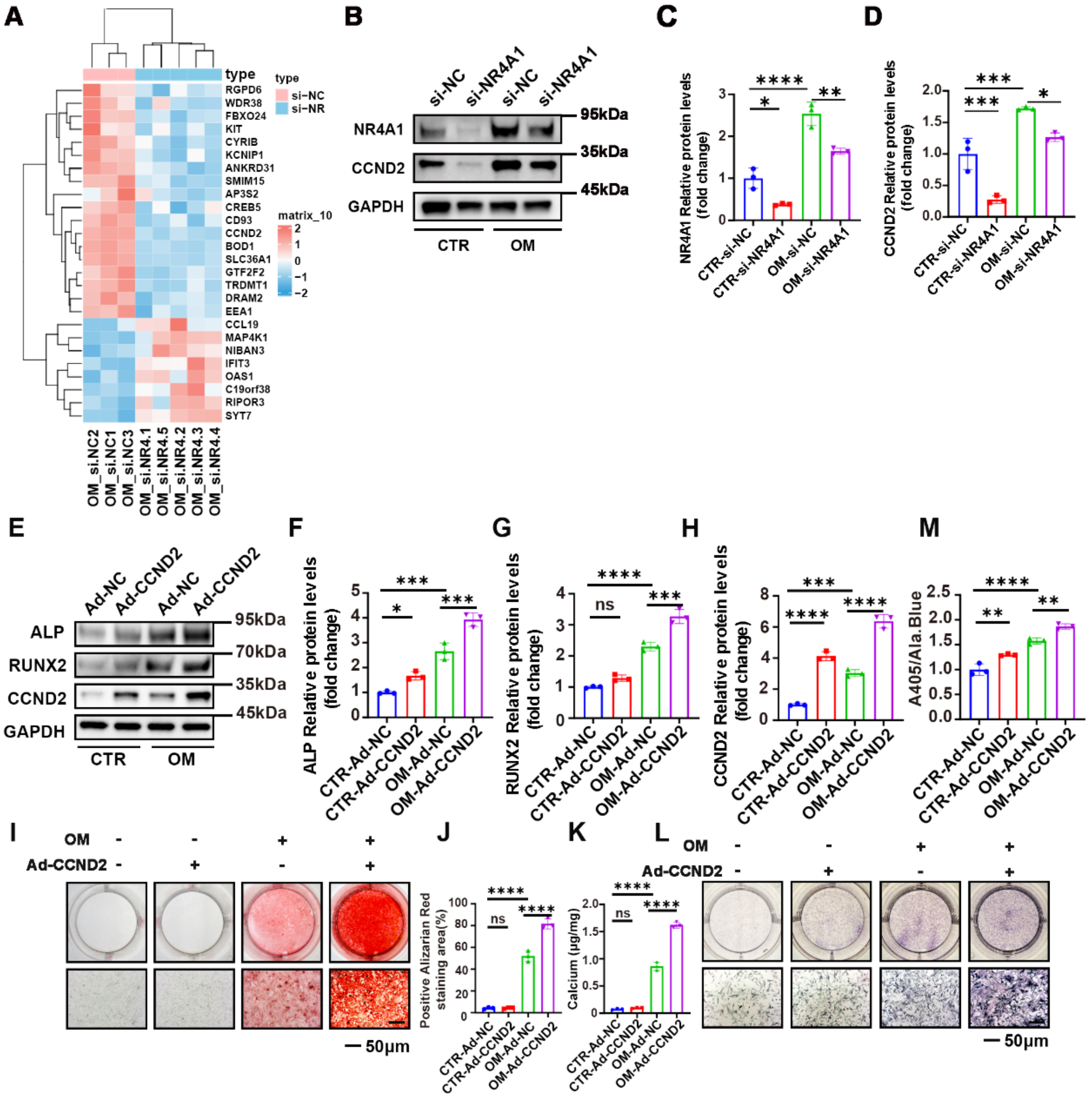

Figure S3. (A) The heatmap visualizes the expression patterns of selected differentially expressed genes between the two groups (B-D) WB analysis of CCND2 expression following NR4A1 silencing (n = 3, each group). (E-H) After infecting VICs with adenovirus carrying CCND2 for 48 hours, the levels of osteogenic differentiation markers, namely ALP and RUNX2, were evaluated by WB and quantitatively analyzed. (I-K) Alizarin Red S staining of VICs after 21 days of osteogenic induction with CCND2 overexpression. (L-M) Representative image of ALP staining of VICs after 7 days of osteogenic induction with CCND2 overexpression (n= 3 per group). Scale bar: 50 μm. Data are shown as means ± SD. ANOVA with Tukey's multiple comparisons test. * $p < 0.05$, ** $p < 0.01$, *** $p < 0.001$, and **** $p < 0.0001$.

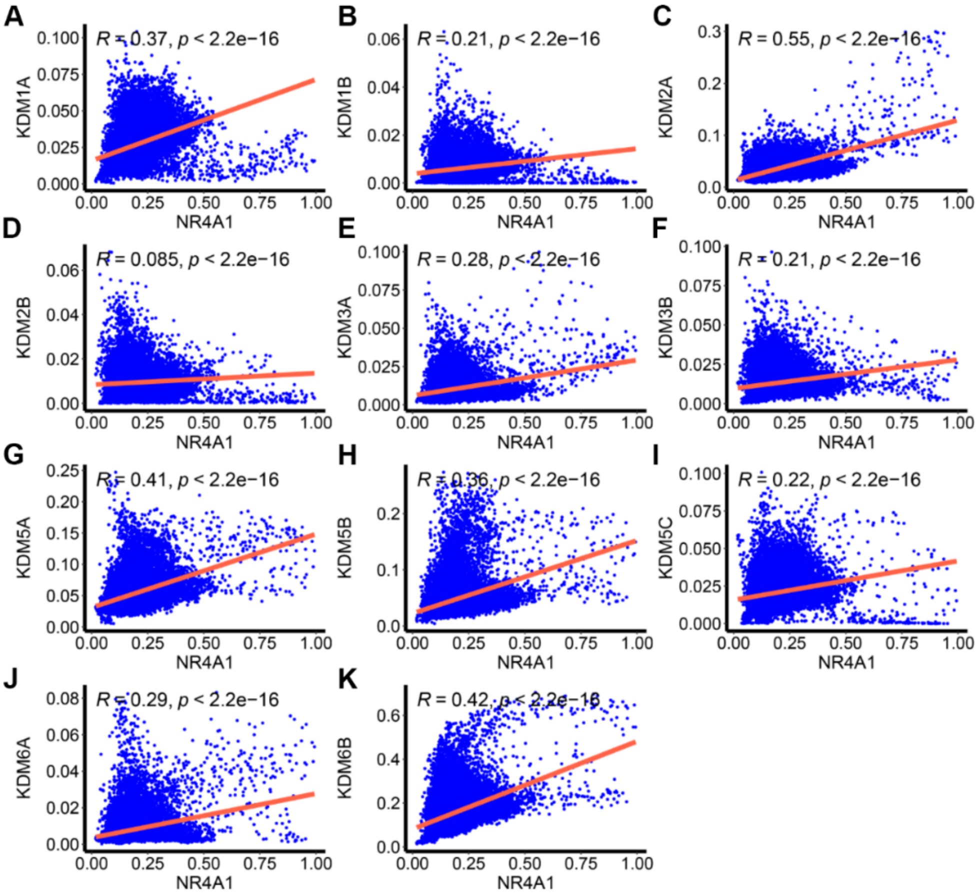

Figure S4. (A-K) Single-cell analysis of 11 common histone demethylases demonstrated marked differences between normal cells and CAVs, along with a correlation to NR4A1 expression.

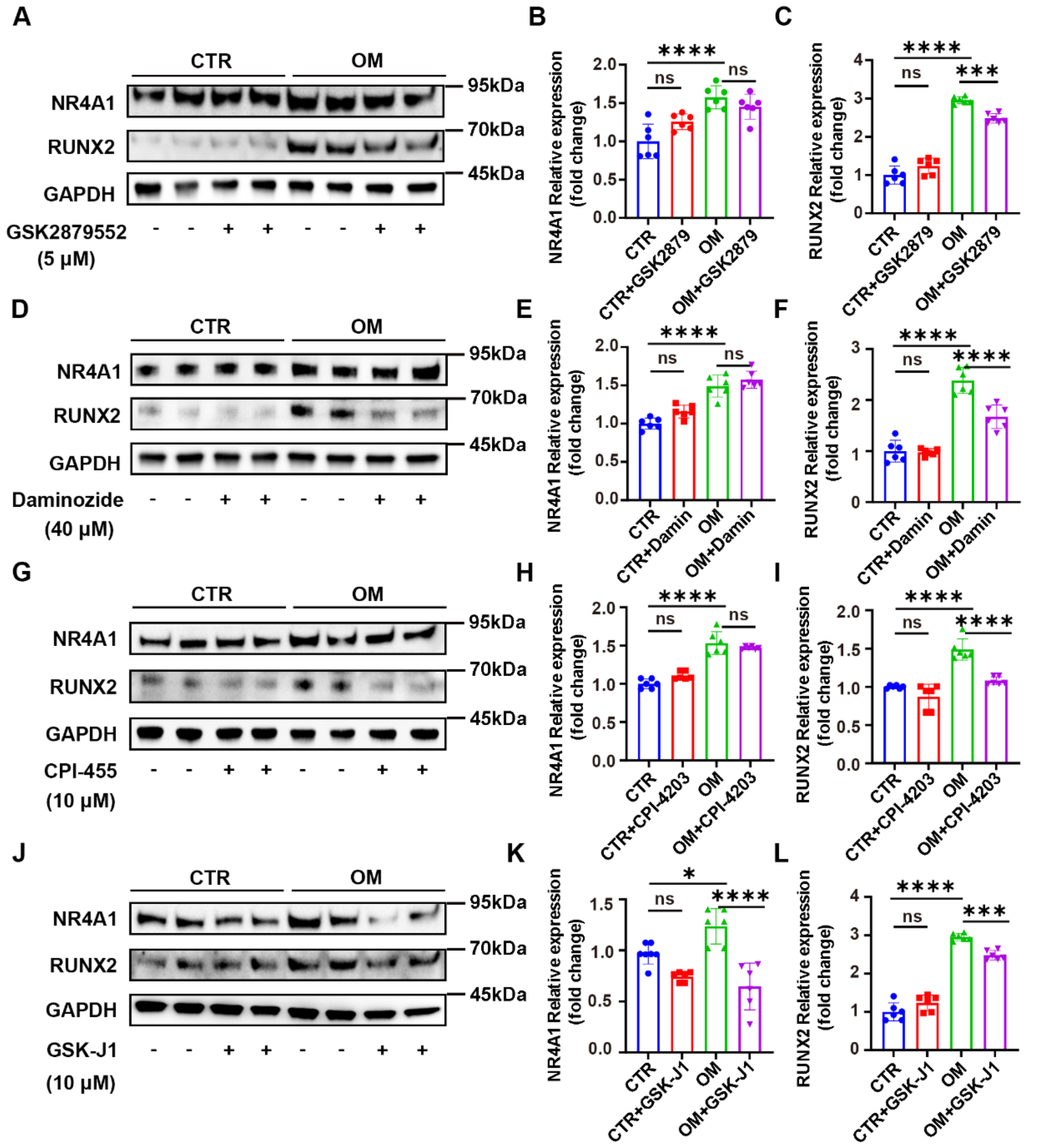

Figure S5. (A-L) Four specific demethylase inhibitors were employed to investigate their effect on the regulation of NR4A1 expression (n = 6, each group). Data are shown as means ± SD. ANOVA with Tukey's multiple comparisons test. * $p < 0.05$, ** $p < 0.01$, *** $p < 0.001$, and **** $p < 0.0001$.

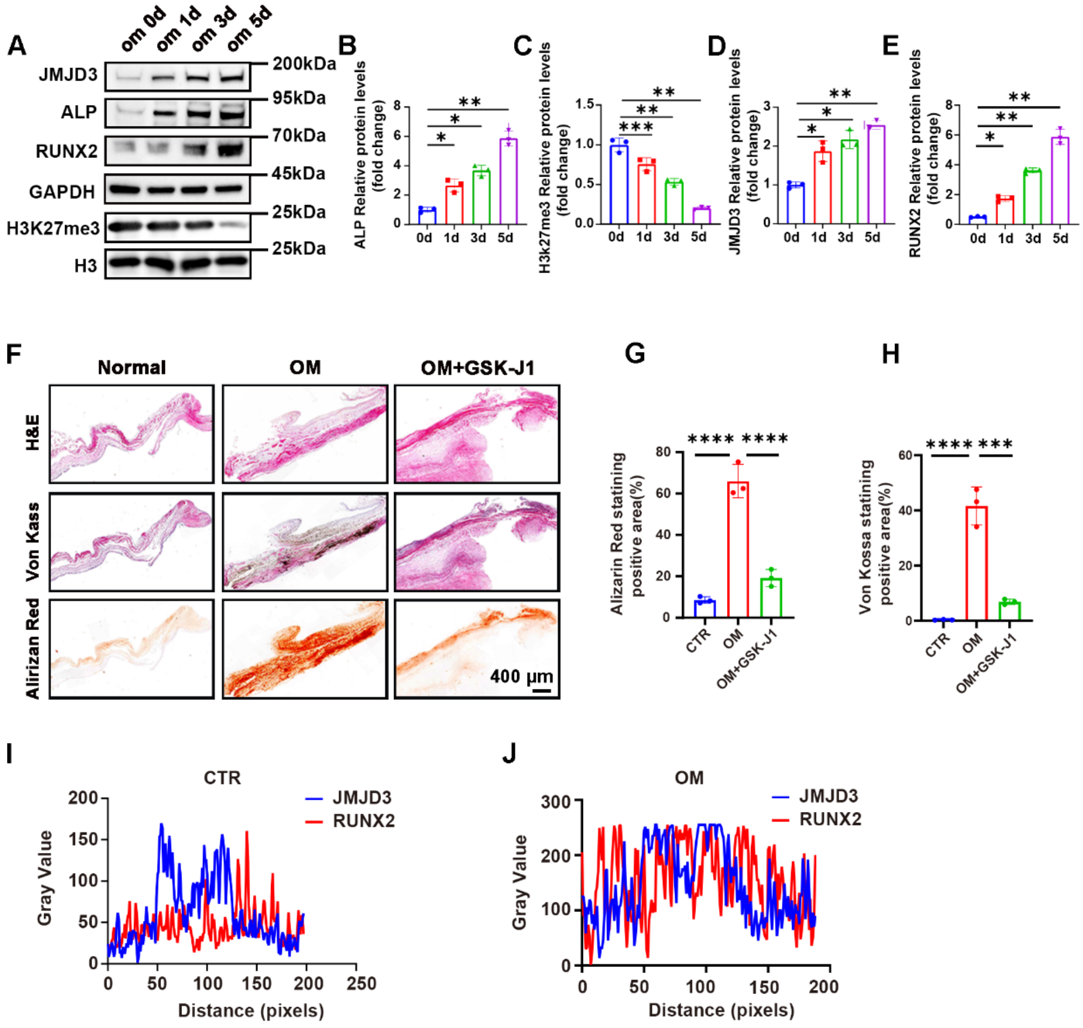

Figure S6 (A-E) The protein levels of JMJD3 and H3K27ME3 following 0, 1, 3, and 5 days of OM stimulation (n = 3, each group). (F-H) Histological analysis of aortic valve tissues treated with GSK-J1 and osteogenic medium, stained with H&E, Von Kossa, and Alizarin Red (n = 3, each group). Scale bar: 400 μm. (I-J) Colocalization analysis of JMJD3 and RUNX2. Fluorescence intensity of JMJD3 and RUNX2 was measured at the positions indicated by the yellow and blue lines. Scale bar: 400 μm. Data are shown as means ± SD. ANOVA with Tukey's multiple comparisons test. * $p < 0.05$, ** $p < 0.01$, *** $p < 0.001$, and **** $p < 0.0001$.

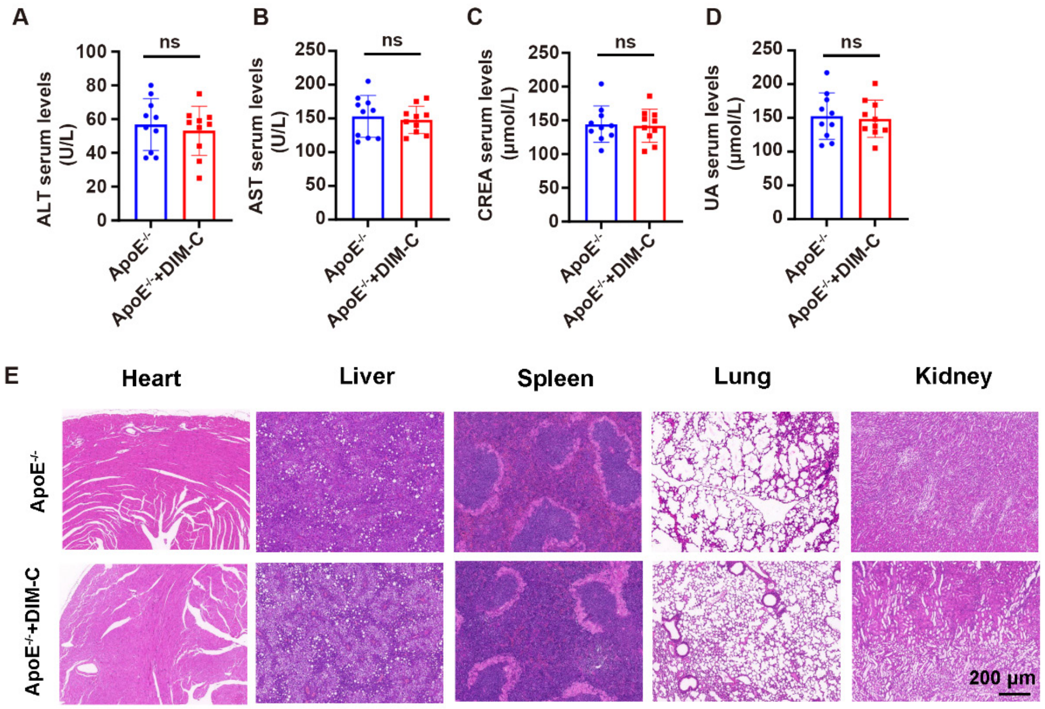

Figure S7 (A-D) Levels of liver and kidney condition indexes in the serum, including ALT, AST, CREA, UA in ApoE$^{-/-}$ mice and ApoE$^{-/-}$ mice treated with DIM-C (n = 10 per group). (E) HE staining of heart, liver, spleen, lung and kidney in ApoE$^{-/-}$ and ApoE$^{-/-}$ + DIM-C mice Scale bar: 200 μm. Data are shown as means ± SD. ANOVA with Tukey's multiple comparisons test. * $p < 0.05$, ** $p < 0.01$, *** $p < 0.001$, and **** $p < 0.0001$.
